# Exogenous Tebuconazole and Trifloxystrobin Regulates Reactive Oxygen Species Metabolism Toward Mitigating Salt-Induced Damages in Cucumber Seedling

**DOI:** 10.3390/plants8100428

**Published:** 2019-10-18

**Authors:** Sayed Mohammad Mohsin, Mirza Hasanuzzaman, M. H. M. Borhannuddin Bhuyan, Khursheda Parvin, Masayuki Fujita

**Affiliations:** 1 Laboratory of Plant Stress Responses, Department of Applied Biological Sciences, Faculty of Agriculture, Kagawa University, Miki-Cho, Kita-Gun, Kagawa 761-0795, Japan; mohsinsau.ac@gmail.com (S.M.M.); razon_sau@yahoo.com (M.H.M.B.B.); hirasau@gmail.com (K.P.); 2 Department of Plant Pathology, Faculty of Agriculture, Sher-e-Bangla Agricultural University, Sher-e-Bangla Nagar, Dhaka-1207, Bangladesh; 3 Department of Agronomy, Faculty of Agriculture, Sher-e-Bangla Agricultural University, Sher-e-Bangla Nagar, Dhaka-1207, Bangladesh; 4 Bangladesh Agricultural Research Institute, Joydebpur, Gazipur 1701, Bangladesh; 5 Department of Horticulture, Faculty of Agriculture, Sher-e-Bangla Agricultural University, Sher-e-Bangla Nagar, Dhaka-1207, Bangladesh

**Keywords:** fungicides, biocatalyst, reactive oxygen species, antioxidant defense, osmotic stress

## Abstract

The present study investigated the role of tebuconazole (TEB) and trifloxystrobin (TRI) on cucumber plants (*Cucumis sativus* L. cv. Tokiwa) under salt stress (60 mM NaCl). The cucumber plants were grown semi-hydroponically in a glasshouse. Plants were exposed to two different doses of fungicides (1.375 µM TEB + 0.5 µM TRI and 2.75 µM TEB + 1.0 µM TRI) solely and in combination with NaCl (60 mM) for six days. The application of salt phenotypically deteriorated the cucumber plant growth that caused yellowing of the whole plant and significantly destructed the contents of chlorophyll and carotenoids. The oxidative damage was created under salinity by increasing the contents of malondialdehyde (MDA), hydrogen peroxide (H_2_O_2_), and electrolytic leakage (EL) resulting in the disruption of the antioxidant defense system. Furthermore, in the leaves, stems, and roots of cucumber plants increased Na^+^ content was observed under salt stress, whereas the K^+^/Na^+^ ratio and contents of K^+^, Ca^2+^, and Mg^2+^ decreased. In contrast, the exogenous application of TEB and TRI reduced the contents of MDA, H_2_O_2_, and EL by improving the activities of enzymatic and non-enzymatic antioxidants. In addition, ion homeostasis was regulated by reducing Na^+^ uptake and enhanced K^+^ accumulation and the K^+^/Na^+^ ratio after application of TEB and TRI. Therefore, this study indicates that the exogenous application of TEB and TRI enhanced salt tolerance in cucumber plants by regulating reactive oxygen species production and antioxidant defense systems.

## 1. Introduction

Climate changes, especially global warming and environmental calamities, severely affect plant productivity worldwide. It also leads to the development of various abiotic stresses, such as salinity, metal/metalloids toxicity, drought, low and high temperatures, flooding, atmospheric pollutants, and ultraviolet-radiation [1]. Among them, salinity is a very common threat to reduce the growth, productivity, and yield of crop plants [2].

Salinity causes osmotic stress [3] as well as ionic toxicity [4], which affect morphological, physiological, and biochemical processes of plants [5]. Salinity reduces the rate of photosynthesis and increases the reactive oxygen species (ROS) formation and, ultimately, causes oxidative stress by disrupting the antioxidant defense system [1,6]. Salt stress produces significant amounts of ROS (singlet oxygen, ^1^O_2_, superoxide, O_2_^•−^, hydrogen peroxide, H_2_O_2_, and hydroxyl radical, OH^•^) [7], which are extremely toxic and cause cell damage, lipid peroxidation, protein denaturing, and programmed cell death [8].

To develop salt stress tolerance, plants improved osmotic and ionic balance and ROS detoxification [4,9]. Moreover, plants mitigate osmotic injury by producing different osmolytes (such as proline, glycinebetaine) to regulate the water balance and stabilize the protein and enzyme structures [10,11]. In addition, plants can modulate the antioxidant defense mechanism under stress conditions to detoxify ROS, which is driven by enzymatic and non-enzymatic antioxidants [12,13]. The enzymatic antioxidants are mainly superoxide dismutase (SOD), catalase (CAT), glutathione *S*-transferase (GST), glutathione peroxidase (GPX), ascorbate peroxidase (APX), monodehydroascorbate reductase (MDHAR), dehydroascorbate reductase (DHAR), and glutathione reductase (GR), while non-enzymatic antioxidants are mainly ascorbate (AsA), glutathione (GSH), alkaloids, α-tocopherols, phenolic compounds, and some amino acids [13,14].

Exogenous application of bio-stimulants such as plant growth hormones, trace elements, organic chemicals, signaling molecules, etc., are now the most well-liked technique to mitigate the oxidative stress and develop stress tolerance in plants [15,16]. Fungicides are chemicals, which are normally used to control plant diseases by destroying disease-causing fungi. However, some fungicides such as triazole and strobilurin, are able to provide protection against biotic and abiotic stresses. The strobilurins fungicides inhibit the growth of fungi by blocking the electron transport at the cytochrome-bc_1_ complex during the respiration in mitochondria [17]. They also prompt a positive effect on plant growth and physiology by interacting with the transfer of electrons in mitochondria [18]. Strobilurins application increased the yields of grain, kernel weights, and contents of protein related to a delay of flag leaf senescence [19], as well as increased abiotic stress tolerance [20]. Triazole is another fungicide that has high affinity to increase the activity of cytochrome-P450 oxidase enzyme in fungi [21], which is responsible for demethylation of ergosterol precursor 24-methylenedihydrolanosterol via several oxidation processes [22]. However, ergosterol is a vital membrane component in most of the fungi, and inhibition of sterol synthesis leads to failure of membrane stability and ultimate fungal cell death [22]. Apart from this, several reports also showed the effect of triazole fungicides in plant physiology [23] by improving plant growth and biomass, chlorophyll (chl) content, and the activities of antioxidant enzymes (SOD, CAT, and APX) [23,24].

Cucumber (*Cucumis sativus* L.), which is widely popular and an economically important fruit vegetable crop, contains a medicinal value, and is the source of raw material for various industries [25]. It is a highly salt-sensitive crop [26,27]. Salinity reduces the growth and production of the cucumber [28,29,30]. Exogenous application of triazole [31] and strobilurin [32] fungicides can alleviate salt stress, but the exact mechanism is still unknown. There are hardly any studies reporting the strobilurin and triazole fungicides effect on plant physiological and biochemical mechanisms. Thus, the experiment was undertaken to investigate the role of tebuconazole(TEB, triazole fungicide) and trifloxystrobin(TRI, strobilurin fungicide) in relation to salt stress tolerance by modulating the antioxidant system of cucumber plants.

## 2. Results

### 2.1. Plant Growth

**Figure 1:**
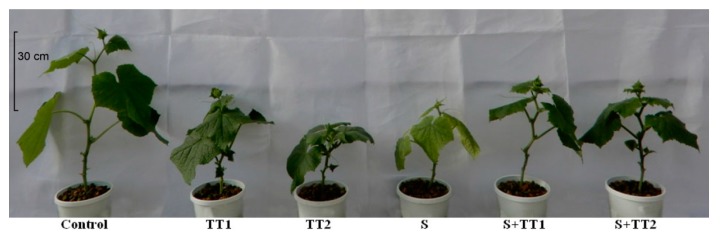
Phenotypic appearance of cucumber plants under different treatments. (TT1, 1.375 µM TEB + 0.5 µM TRI; TT2, 2.75 µM TEB + 1.0 µM TRI; S, 60 mM NaCl; respective treatments were applied on 50–day old plants for six days).

Phenotypic appearance clearly indicated that salinity inhibited the growth and development of the cucumber plant (Figure 1). Plants treated with salt caused yellowing of the whole plant, whereas the supplementation of TEB and TRI improved the phenotypic appearance of the cucumber plant by reducing the salt-induced damage.

Salt treatment reduced the height, leaf number, and internodes’ length (Table 1) of cucumber plants. Salt stress also decreased the fresh weight (FW) and dry weight (DW) of leaves and roots (Table 2). Compared with the control, salinity decreased the plant height, number of leaves, and length of internodes by 61%, 17%, and 34%, respectively (Table 1). Similarly, it also reduced the leaf FW, leaf DW, root FW, and root DW by 30%, 44%, 44%, and 46%, respectively. This is in contrast with the control (Table 2). No significant difference was found after application of both doses of TEB and TRI on salt-treated plants for plant height and number of leaves, but a significant difference was shown for the length of internodes, leaf FW, leaf DW, root FW, and root DW at a high dose of TEB and TRI, compared to stress plant only.

### 2.2. Photosynthetic Pigments

**Figure 2:**
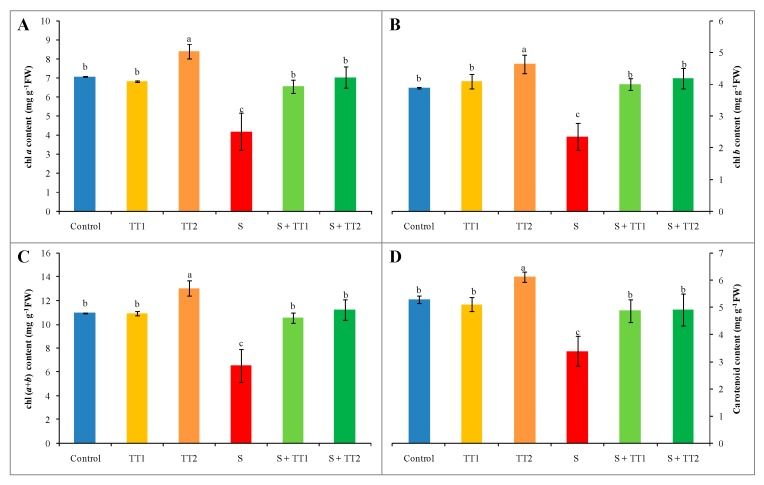
Effect of TEB and TRI on photosynthetic pigment contents: chl *a* (**A**), chl *b* (**B**), chl (*a+b*) (**C**), and carotenoid (**D**) in leaf of 50–day-old cucumber plants under salt stress for six days. In this case, TT1, TT2, and S indicate 1.375 µM TEB+0.5 µM TRI, 2.75 µM TEB+1.0 µM TRI, and 60 mM NaCl, respectively. Means (±SD) were calculated from three replicates for each treatment. Bars with different letters are significantly different at *p* ≤ 0.05 by applying the Fisher’s LSD test.

Salt stress significantly destroyed the photosynthetic pigments of cucumber plants, which was evident from the content of chl and carotenoid (car) reduction. Compared to the control, the content of chl *a* and *b* were reduced by 41% and 39%, respectively. Under salt stress, therefore, chl (*a+b*) content was also reduced by 40%. Salt treatment also reduced the car content by 36% compared to the control. Alternatively, the application of both low and high doses of TEB and TRI significantly recovered the chl and car content (Figure 2A–D).

### 2.3. MDA and ROS Production

**Figure 3:**
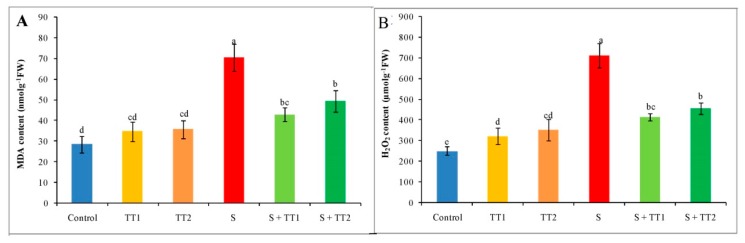
Effect of TEB and TRI on MDA and ROS production, MDA content (**A**), and H_2_O_2_ content (**B**) in leaves of 50–day-old cucumber plants under salt stress for 6 days. In this case, TT1, TT2, and S indicate 1.375 µM TEB+0.5 µM TRI, 2.75 µM TEB+1.0 µM TRI, and 60 mM NaCl, respectively. Means (±SD) were calculated from three replicates for each treatment. Bars with different letters are significantly different at P ≤ 0.05 by applying the Fisher’s LSD test.

Salt-treated cucumber plants were considerably damaged by oxidative stress signified by the content of the lipid peroxidation component (malondialdehyde, MDA content) and production of ROS (H_2_O_2_ content). Under stress conditions, MDA content increased by 147% in contrast with the control. Apart from this, salt treatment increased H_2_O_2_ content by 184%, compared with the control. On the contrary, exogenous application of TEB and TRI at both low and high doses significantly mitigated the oxidative damage by declining the content of MDA and H_2_O_2_, where a low dose showed a better result than a high dose of TEB and TRI (Figure 3A,B).

### 2.4. Antioxidant Non-Enzymatic

**Figure 4:**
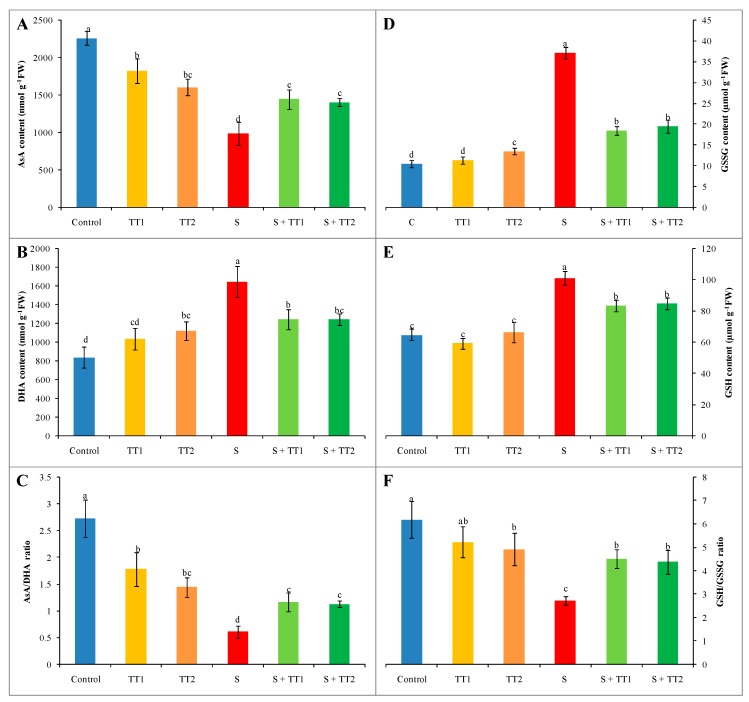
Effect of TEB and TRI on the non-enzymatic antioxidant. AsA content (**A**), DHA content (**B**), AsA/DHA ratio (**C**), GSSG content (**D**), GSH content (**E**), and GSH/GSSG ratio (**F**) in leaf of 50–day-old cucumber plants under salt stress for six days. Here, TT1, TT2, and S indicate 1.375 µM TEB+0.5 µM TRI, 2.75 µM TEB+1.0 µM TRI and 60 mM NaCl, respectively. Means (±SD) were calculated from three replicates for each treatment. Bars with different letters are significantly different at *p* ≤ 0.05 when applying the Fisher’s LSD test.

In comparison to the control, the AsA content decreased by 56% while dehydroascorbate (DHA) content increased by 96% in response to salt stress, which resulted in a 78% reduction of the AsA/DHA ratio. On the contrary, the application of either doses of TEB and TRI could increase in AsA content and the AsA/DHA ratio in a stressed plant compared to the stressed group. However, the DHA content was markedly reduced by the application of both doses of TEB and TRI in salt exposed plants than stressed plants only (Figure 4A–C).

Salt stress increased the GSH and oxidized glutathione (GSSG) content by 56% and 253%, respectively. The concomitant reduction of the GSH/GSSG ratio was about 56% compared to the control. However, TEB and TRI treatments improved the GSH/GSSG ratio by reducing both GSSG and GSH contents in salt-exposed plants, in contrast with salt stress alone (Figure 4D–F).

### 2.5. Antioxidant Enzymes

**Figure 5:**
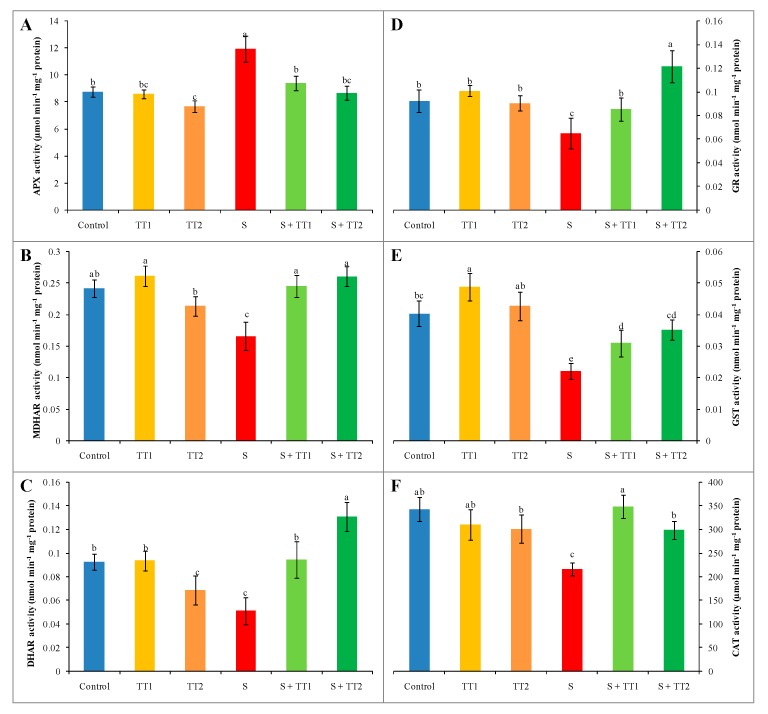
Effect of TEB and TRI on AsA-GSH pathway enzymes. APX (**A**), MDHAR (**B**), DHAR (**C**), GR (**D**), GST (**E**), and CAT (**F**) activity in leaf of 50–day-old cucumber plants under salt stress for six days. Here, TT1, TT2, and S indicate 1.375 µM TEB+0.5 µM TRI, 2.75 µM TEB+1.0 µM TRI, and 60 mM NaCl, respectively. Means (±SD) were calculated from three replicates for each treatment. Bars with different letters are significantly different at *p* ≤ 0.05 by applying the Fisher’s LSD test.

Salt treatment enhanced the APX activity by 36%, whereas MDHAR, DHAR, and GR activity declined by 31%, 45%, and 30%, respectively; compared to the control. On the other hand, the application of TEB and TRI at both low and high doses in salt-treated cucumber plants declined APX activity by 21% and 27%, respectively. However, it increased the activity of MDHAR by 48% and 57%, DHAR by 84% and 155%, and GR by 32% and 87%, respectively; compared with only stress plants (Figure 5A–D). Furthermore, salt stress reduced the activity of CAT and GST by 37% and 45%, respectively, in contrast with the control. However, the use of TEB and TRI of low and high doses with salt increased the CAT activity by 61% and 38% and GST activity by 40% and 59%, respectively; compared with stress plants only (Figure 5E,F).

### 2.6. Electrolytic Leakage

**Figure 6:**
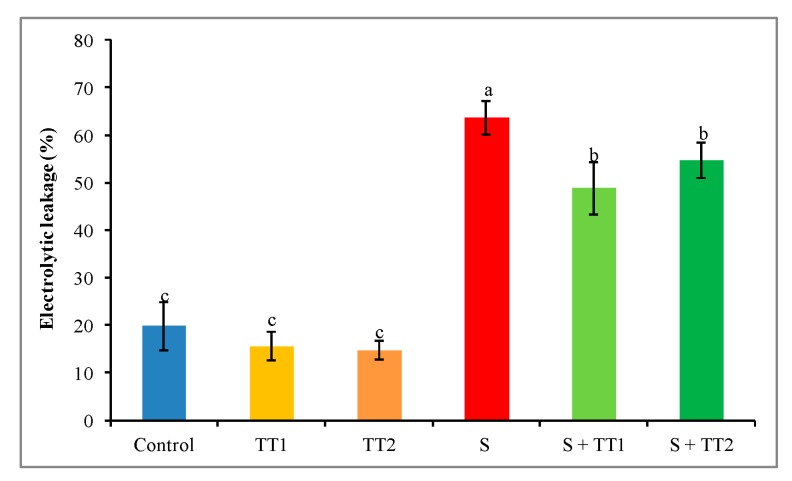
Effect of TEB and TRI on electrolytic leakage in leaves of 50–day-old cucumber plants under salt stress for six days. Here, TT1, TT2, and S indicate 1.375 µM TEB+0.5 µM TRI, 2.75 µM TEB+1.0 µM TRI, and 60 mM NaCl, respectively. Means (±SD) were calculated from three replicates for each treatment. Bars with different letters are significantly different at *p* ≤ 0.05 when applying the Fisher’s LSD test.

A remarkable increase in electrolyte leakage (EL) was observed in cucumber plants under salt stress. In contrast to the control, salt treatment increased EL by 222%, while exogenous application of TEB and TRI at either dose significantly reduced the EL. However, a low dose showed a better result than a high dose of TEB and TRI (Figure 6).

### 2.7. Ion Homeostasis

**Figure 7:**
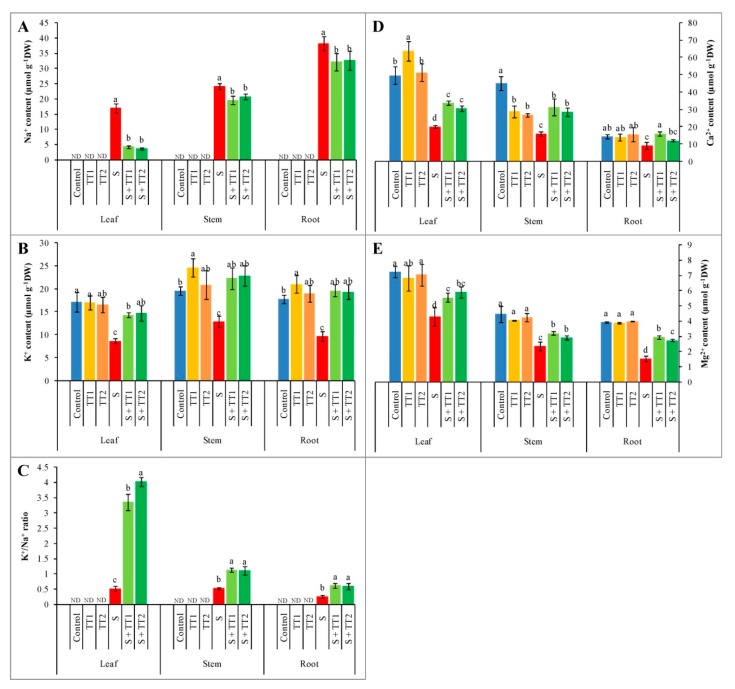
Effect of TEB and TRI on ion homeostasis. Na^+^ contents (**A**), K^+^ contents (**B**), K^+^/Na^+^ ratio (**C**), Ca^2+^ content (**D**), and Mg^2+^ content (**E**) in the leaf, stem, and root of 50–day-old cucumber plants under salt stress for six days. Here, TT1, TT2, and S indicate 1.375 µM TEB+0.5 µM TRI, 2.75 µM TEB+1.0 µM TRI, and 60 mM NaCl, respectively. Means (±SD) were calculated from three replicates for each treatment. Bars with different letters are significantly different at *p* ≤ 0.05 when applying the Fisher’s LSD test.

Ion homeostasis was disrupted in the leaves, stems, and roots of salt-treated cucumber plants by increasing the accumulation of Na^+^ and decreasing the K^+^/Na^+^ ratio and K^+^ uptake, compared to the control. On the contrary, the exogenous application of TEB and TRI at low and high doses on salt-treated cucumber plants decreased Na^+^ accumulation, and enhanced the K^+^/Na^+^ ratio and the uptake of K^+^ in the leaves, stems, and roots that were observed when compared to salt-treated plants alone. In contrast to stressed plants, the K^+^/Na^+^ ratio increased after the application of TEB and TRI at low and high doses by 550% and 679% in leaves, 113% and 107% in stems, and 141% and 135% in roots, respectively (Figure 7A–C). Furthermore, the accumulation of Ca^2+^ was reduced in the leaves, stems, and roots of salt-treated plants by 59%, 40%, and 36%, respectively; compared to the control. Similarly, salt stress reduced the Mg^2+^ accumulation in the leaves, stems, and roots by 41%, 47%, and 61%, respectively, in contrast with the control plants. Moreover, the exogenous application of both low and high doses TEB and TRI increased Ca^2+^ and Mg^2+^ content in the leaves, stems, and roots of salt-exposed cucumber plants, compared to salt stress alone (Figure 7D,E).

## 3. Discussion

This study was aimed at understanding the antioxidative role of TEB and TRI to mitigate the salinity-induced oxidative damages in cucumber plants. The introductions of triazoles and strobilurins fungicides have been exploring the new concept, which has not only the disease control ability but also has positive effects on plant physiology [33]. In this study, salt stress reduced the plant growth and biomass, which might be due to the higher accumulation of Na^+^ [34], and the reduction of the photosynthetic pigment content [35]. A similar loss of plant growth and biomass was reported by Wang et al. [36] and Wu et al. [37] in stressed cucumber plants. On the other hand, the application of TEB and TRI in salt-treated plants restored the growth parameters. This might be due to the improvement of ion homeostasis. Several reports also found that triazole fungicides enhanced plant growth and biomass under salinity stress [38,39]. However, the sole treatment of TEB and TRI showed a negative effect on plant height. This might be due to the activity of triazoles on isoprenoid pathway, which inhibits gibberellic acid synthesis [40].

The complex of pigment-protein becomes destabilized under high salt concentration, which increases the activity of the chlorophyllase enzyme and produces a higher amount of ROS, thus inhibit photosynthetic pigment synthesis [2]. In the present study, we observed that the salt-treated plants reduced the content of chl and car. However, using TEB and TRI with salt significantly increased the chl and car content. The content of chl and car improvement might be due to increasing the production of cytokinin after applying tebuconazole, which stimulates the biosynthesis of the photosynthesis pigments [41].

The crucial effect of salt stress is the overproduction of ROS, which increases lipid peroxidation and inhibits ROS-scavenging enzymes’ activities [2,14]. In the present investigation, the cucumber plants exposed to salt showing an excessive production of H_2_O_2_ might be due to the destruction of the membrane properties by enhancing lipid peroxidation (MDA content). Damaging the cell membrane indicates the production of salt-induced oxidative stress. These results supported by previously published reports that salinity enhanced the permeability of the membrane by improving the content of MDA in cucumber plants [42,43]. On the other hand, exogenous TEB and TRI reduced salt-induced oxidative damage in cucumber plants by inhibiting the overproduction of ROS and lipid peroxidation. A number of reports found that exogenous application of triazole and strobilurin fungicide reduced the overproduction of ROS to mitigate lipid peroxidation of the cell membrane [44,45,46]. 

Ascorbate and GSH are non-enzymatic antioxidants, which play a vital role to defend plant cells and biomolecules from oxidative stress by quenching ROS [14]. To maintain the cellular redox state, AsA/DHA and GSH/GSSG ratios are more important under abiotic stress [47]. The antioxidant, AsA, can directly react and quench of ROS [14] and GSH regulates the GPX and GST enzymes’ activity to scavenge ROS and create plant stress tolerance [48]. In the present study, AsA content was reduced by salinity while DHA content increased. Therefore, salinity stress reduced the AsA/DHA ratio, which indicated the overproduction of ROS and enhanced oxidative damage. Under salt stress, lower AsA content and AsA/DHA ratio was also found by Hasanuzzaman et al. [49]. In contrast, the exogenous TEB and TRI improved the content of the AsA and AsA/DHA ratio, while the content of DHA declined with increasing activity of MDHAR and DHAR. A similar report published by Akbari et al. [39], who found that the uses of hexaconazole in canola leaves increased the AsA content under salt stress. To improve plant tolerance under salt stress, GSH plays a vital role by scavenging ROS and regenerating the content of AsA [50]. In the present study, salt treatment enhanced the content of GSSG and GSH, which resulted in the decrease in the GSH/GSSG ratio. Similar results also reported by Nahar et al. [15], where salinity improved GSSG content, while the GSH/GSSG ratio decreased. On the other hand, exogenous TEB and TRI decreased the content of GSSG and GSH, where the GSH/GSSG ratio was enhanced with increasing GR activity. The results suggested that TEB and TRI improved the AsA and GSH content under salt stress and related findings have been reported by Sankar et al. [51] in the *Arachis hypogaea* L. plant and Akbari et al. [39] in the *Brassica napus* L. plant.

The AsA-GSH cycle pathway enzymes (APX, MDHAR, DHAR, and GR) efficiently work together with non-enzymatic antioxidants (AsA and GSH) to scavenge ROS and reproduce AsA and GSH [47]. In the present study, the activity of APX significantly increased, where MDHAR, DHAR, and GR activity reduced under salt stress. Moreover, under salinity, lower content of AsA was found, which might be due to higher APX activity and lower activity of MDHAR and DHAR. This result was corroborated by Hasanuzzaman et al. [52]. The APX detoxify the H_2_O_2_ to H_2_O using AsA and produce MDHA and DHA where, MDHAR and DHAR are responsible for restoring AsA from MDHA and DHA with the help of nicotinamide adenine dinucleotide phosphate (NADPH) and GSH [6]. On the other hand, the activity of MDHAR and DHAR significantly increased in TEB and TRI-treated plants under salt stress, which might be responsible for the increase of AsA content. In addition, exogenous TEB and TRI treatment also increased the GR activity and regulated the GSH/GSSG ratio. 

In our investigation, lower CAT activity was found under salt stress, which increased the production of H_2_O_2_ and a similar result was also found by Hasanuzzaman et al. [13]. Regardless, cucumber plants were treated with TEB and TRI with salt, which increased the CAT activity by reducing H_2_O_2_ production. This result is supported by Liang et al. [45]. The enzyme GST has multi-functional activities, which can scavenge H_2_O_2_ by using GSH and has the ability to detoxify the xenobiotic substances [53]. In this study, the activity of GST was reduced under salt stress, which is supported by Hasanuzzaman et al. [13]. In contrast, exogenous TEB and TRI improved the activity of GST, which might help detoxify H_2_O_2_ in salt-treated cucumber plants. 

Cell membrane injury can be easily identified by EL under stress conditions. It is the indicator of cell membrane integrity [54]. In the present study, salt-stress significantly increased the EL, which hampered the cell membrane integrity. This was evident from higher lipid peroxidation (MDA). Similar results were also reported by Zhu et al. [55], who found salinity-imposed cell membrane damage of cucumber plants by creating higher EL. However, exogenous TEB and TRI reduced salt-induced damage by inhibiting the higher EL. Decreased EL indicated the reduction of membrane damage and lower lipid peroxidation, which resulted in membrane stability improved. Arivalagan et al. [56] also found that exogenous propiconazole regulated cell membrane integrity by reducing EL. 

Under a high salt concentration, the initial response of plants is Na^+^-induced K^+^ efflux. Higher Na^+^ accumulation under salinity condition reduces the K^+^/Na^+^ ratio and disrupts ion homeostasis [57]. In our study, under the salt condition, higher content of Na^+^ was found in all observed plant parts, where the K^+^ content was lower. This resulted in a decreased K^+^/Na^+^ ratio. The higher accumulation of Na^+^ was found in the roots when compared to the stems and leaves. Similarly, Wu and Wang [58] reported higher Na^+^ concentration in rice plant roots than shoots as well as a lower K^+^/Na^+^ ratio under salinity. The higher Na^+^ concentration and lower K^+^ was also found under salt stress, which was reported by Kaya et al. [59] and Stepien and Kobus [60]. However, exogenous TEB and TRI reduced the uptake of Na^+^ while increasing the K^+^ uptake and K^+^/Na^+^ ratio in leaves, stems, and roots of cucumber plants under salt stress. Hajihashemi et al. [61] also observed that the application of paclobutrazol (PBZ) decreased the accumulation of Na^+^ in the wheat plant, while enhancing the contents of K^+^, P, and N under salt stress. In our investigation, salt treatment reduced Ca^2+^ and Mg^2+^ content in all observed plant parts, which might be due to the Na^+^ activity to displace the Ca^2+^ and Mg^2+^. Similar results were found by Rahman et al. [4] in rice plants under salt stress. However, the application of TEB and TRI improved the Ca^2+^ and Mg^2+^ content in all parts of cucumber plants that were exposed to salinity.

## 4. Materials and Methods

### 4.1. Plant Materials and Test Conditions

Healthy and uniform cucumber (*Cucumis sativus* L. cv. Tokiwa) seeds were selected to perform this experiment. Cucumber plants were grown in a glasshouse at normal light conditions on 25–28 °C air temperature and relative humidity of 60–70%. Seedling plug trays were used for sowing the seeds, which filled with the mixture of vermiculite and peat at the ratio of 1:2 (*v*/*v*). After 29 days of seed sowing, the first true leaf was developed and then seedlings were transferred to plastic pots using grow rock and half-strength Hoagland solution was used as nutrients [62]. After every three days, nutrient solutions were changed during the growing period. Twenty-one days after transplanting, the plants were exposed to salt (60 mM NaCl) and fungicides (1.375 µM TEB + 0.5 µM TRI, and 2.75 µM TEB + 1.0 µM TRI) solely and in combination for the next 6 days. Un-treated plants were grown with nutrient solution only. The experiment was arranged in a completely randomized design (CRD) and each treatment was replicated three times. 

### 4.2. Salt Toxicity Symptoms and Growth Parameters

Plant growth was determined by measuring plant height, number of leaves plant^−1^, internodes length, and FW and DW of leaf and root of the plants. The height of the plant was observed from the shoot base to the tip of the top leaf. After harvest, the leaf and root were weighed for FW determination and then dried at 70 °C for 48 h to measure DW.

### 4.3. Determination of Photosynthetic Pigment Content

Photosynthetic pigment (chl and car) was measured according to Lichtenthaler [63]. Leaves were cut into smaller pieces and placed in small centrifuge tubes containing 100% ethanol. The samples were heated in a water bath at 60 °C. Thereafter, the absorbance of each sample was cooled and measured spectrophotometrically at 664, 648, and 470 nm to calculate chl *a*, chl *b*, and car content. 

### 4.4. Determination of Malondialdehyde Content

The content of MDA was observed following the technique of Heath and Packer [64] with modification from Hasanuzzaman et al. [16]. Trichloroacetic acid (TCA) was used to homogenize the fresh leaves and centrifuged at 11,500× *g*. The thiobarbituric acid (TBA) reagent was added with leaf extracts and incubated in a hot water bath. Thereafter, the mixture was quickly chilled to stop the reaction and centrifuged again at 11,500× *g*. The optical absorbance was observed at 532 nm, and corrected at 600 nm.

### 4.5. Observation of H_2_O_2_ Content

Hydrogen peroxide content was observed according to Hossain et al. [65]. Leaves were homogenized with 5% (*w*/*v*) TCA, and centrifuged at 11,500× *g*. After centrifuging, the leaf extract was mixed with K-P buffer and 1 M KI. A standard curve was used to calculate the H_2_O_2_ content after collecting the absorbance at 390 nm.

### 4.6. Observation of Ascorbate and Glutathione Content

Fresh leaves were extracted with 5% (*w*/*v*) TCA, and centrifuged at 11,500× *g*. Total ascorbate and DHA contents were measured following the method of Lechno et al. [66]. For total ascorbate determination, CuSO_4_.5H_2_O was used to oxidize AsA. Therefore, the reaction mixture (dinitrophenyl hydrazine and thiourea dissolved in diluted H_2_SO_4_) was added and incubated for 3 h at 37 °C. Ice-cold H_2_SO_4_ was used to stop the reaction. For the DHA measurement, DW was used instead of CuSO_4_.5H_2_O. The total ascorbate and DHA was observed spectrophotometrically at 520 nm, and known concentrations of AsA were used to prepare a standard curve. The content of AsA was measured by subtracting of DHA from the total ascorbate.

Glutathione was determined following the method of Law et al. [67]. Leaf extract was mixed with K-P buffer (pH 7.0) and DW to be neutralized for total glutathione whereas 2-vinylpyridine was used instead of DW for GSSG. Standard curves were prepared using known concentrations of GSH and GSSG for determining total glutathione and GSSG content, respectively, and measured at 412 nm. The content of GSH was calculated by subtracting GSSG from total glutathione.

### 4.7. Protein Quantification

Protein concentration was determined according to Bradford [68]. Bovine serum albumin (BSA) was used to prepare the standards. 

### 4.8. Enzyme Activity Determination

Leaf tissues were homogenized by using ice-cold extraction buffer containing 50 mM K-P buffer (pH 7.0), 1 mM AsA, 100 mM KCl, 5 mM β-mercaptoethanol, and 10% glycerol (*w*/*v*). Therefore, centrifugation (11,500× *g*) was done, and supernatants were collected separately and further used to determine the enzyme activity assay.

Catalase (CAT; EC: 1.11.1.6) activity was assayed according to Hasanuzzaman et al. [16]. Reduction of absorbance was measured at 240 nm caused by the H_2_O_2_ degradation in the reaction mixture containing 50 mM K-P buffer (pH 7.0), 15 mM H_2_O_2,_ and enzyme solution. Catalase activity was computed using the extinction coefficient 39.4 M^−1^ cm^−1^.

Ascorbate peroxidase (APX, EC: 1.11.1.11) activity was measured according to Nakano and Asada [69], where, the reaction reagent contained 50 mM K-P buffer (pH 7.0), 0.5 mM AsA, 0.1 mM EDTA, 0.1 mM H_2_O_2_, and enzyme solution. Decreased absorbance was observed at 290 nm and the APX activity was estimated using 2.8 mM^−1^ cm^−1^ as the extinction coefficient.

Monodehydroascorbate reductase (MDHAR, EC: 1.6.5.4) activity was estimated following Hossain et al. [70], where the reaction mixture comprised of 50 mM Tris–HCl buffer (pH 7.5), 2.5 mM AsA, AO (0.5 units), 0.2 mM NADPH, and enzyme solution. The absorbance was measured at 340 nm and the activity was computed using 6.2 mM^−1^ cm^−1^ as an extinction coefficient.

The activity of dehydroascorbate reductase (DHAR, EC: 1.8.5.1) was measured according to Nakano and Asada [69], where 50 mM K-P buffer (pH 7.0), 2.5 mM GSH, 0.1 mM DHA, and 0.1 mM EDTA were mixed when making the reaction buffer. The activity was calculated by observing the absorbance at 265 nm and using an extinction coefficient of 14 mM^−1^ cm^−1^.

Glutathione reductase (GR, EC: 1.6.4.2) activity was assayed following Hasanuzzaman et al. [16], where the buffer solution contained 0.1 M K-P buffer (pH 7), 1 mM GSSG, 1 mM EDTA, 0.2 mM NADPH, and enzyme solution. The activity was measured by monitoring the absorbance at 340 nm and estimated using the extinction coefficient 6.2 mM^−1^ cm^−1^.

Glutathione *S*-transferase (GST, EC: 2.5.1.18) activity was estimated according to Hasanuzzaman et al. [49], where the reaction buffer solution was comprised of 100 mM Tris-HCl buffer (pH 6.5), 1 mM 1-chloro-2,4-dinitrobenzene (CDNB), 1.5 mM GSH, and enzyme solution. The increase absorbance was monitored at 340 nm and the activity was estimated using 9.6 mM^−1^ cm^−1^ as the extinction coefficient.

### 4.9. Measurement of Electrolyte Leakage

Electrolytic leakage was measured following the technique of Dionisio-Sese and Tobita [71]. Pieces of leaves were kept into a test tube containing deionized H_2_O and heated at 40 °C. Therefore, test tubes were cooled at room temperature and primary electrical conductivity (EC_1_) was collected using CON 700 EC meter, Eutech Instruments, Singapore. Again, the test tubes were heated using an autoclave and cooled at room temperature and, thus, final electrical conductivity (EC_2_) was observed. To calculate EL, the following formula was used: EL (%) = EC_1_/EC_2_ × 100.

### 4.10. Determination of Mineral Content

The contents of mineral nutrients (Na^+^, K^+^, Ca^2+^, and Mg^2+^) were determined using an atomic absorption spectrophotometer (Shimadzu GFA-7000A, Shimadzu, Japan). The samples of the plant were dried in an oven at 70 °C for 48 h. After drying, 0.1 g of each samples were digested using HNO_3_:HClO_4_ (5:1 *v*/*v*) acid mixture at 70 °C for 48 h. 

### 4.11. Statistical Analysis

Computer-based software XLSTAT v. 2018 [72] was used to analyze the data employing the analysis of variance (ANOVA) technique. Means were compared using Fisher’s least significant difference (LSD) test, where p ≤ 0.05 were considered as significant.

## 5. Conclusions

From the findings of the study, it can be concluded that salinity disrupted the cucumber plant’s growth and physiological activity by the higher accumulation of Na^+^, which was the main cause for the overproduction of ROS and change in ion homeostasis. Salt-induced higher ROS developed oxidative damage and hampered the normal growing mechanisms of cucumber plants. However, the exogenous TEB and TRI could enhance the physiological activity of cucumber plants under salinity, which is linked with the TEB and TRI mediated mitigation of higher ROS by up-regulating the mechanisms of the antioxidant defense. The ion homeostasis also regulated the application of TEB and TRI by increasing the K^+^/Na^+^ ratio under salt condition. Lastly, it is recommended that TEB and TRI fungicide can be used in cucumber plants for better growth by regulating oxidative damage under stress conditions.

## Figures and Tables

**Table 1:** Effect of TEB and TRI on plant height, leaf numbers, and internode length of 50–day-old cucumber plants under salt stress for 6 days. Means (±SD) were calculated from three replicates for each treatment. Values with different letters are significantly different at *p* ≤ 0.05 by applying Fisher’s LSD test. In this case, TT1, TT2, and S indicate 1.375 µM TEB+0.5 µM TRI, 2.75 µM TEB+1.0 µM TRI, and 60 mM NaCl, respectively.

Treatments	Plant Height (cm)	Number of Leaf Plant^−1^	Internodes Length (cm)
Control	50.33 ± 5.50 a	7.66 ± 0.57 b	9.08 ± 1.04 a
TT1	27.00 ± 2.02 b	9.33 ± 1.15 a	4.08 ± 0.52 cd
TT2	19.00 ± 1.05 d	7.66 ± 0.57 b	3.91 ± 0.63 cd
S	19.83 ± 0.76 cd	6.33 ± 0.59 c	6.03 ± 0.46 b
S+TT1	26.00 ± 1.02 b	6.66 ± 0.56 bc	4.55 ± 0.51 c
S+TT2	24.16 ± 1.04 bc	7.33 ± 0.61 bc	3.38 ± 0.42 d

**Table 2:** Effect of TEB and TRI on fresh and dry weights of 50–day-old cucumber plants under salt stress for 6 days. Means ( ± SD) were calculated from three replicates for each treatment. Values with different letters are significantly different at P ≤ 0.05 by applying the Fisher’s LSD test. In this scenario, TT1, TT2, and S indicate 1.375 µM TEB+0.5 µM TRI, 2.75 µM TEB+1.0 µM TRI, and 60 mM NaCl, respectively.

Treatments	Leaf		Root	
	FW (g leaf^−1^)	DW (g leaf^−1^)	FW (g plant^−1^)	DW (g plant^−1^)
Control	2.75 ± 0.157 a	0.39 ± 0.025 ab	11.34 ± 0.56 b	1.14 ± 0.10 b
TT1	2.45 ± 0.136 ab	0.32 ± 0.023 cd	12.38 ± 1.37 b	1.27 ± 0.11 b
TT2	2.61 ± 0.241 a	0.35 ± 0.024 bc	9.38 ± 0.96 c	0.93 ± 0.13 c
S	1.92 ± 0.164 c	0.22 ± 0.029 e	6.32 ± 0.67 d	0.61 ± 0.08 d
S+TT1	2.19 ± 0.152 bc	0.29 ± 0.004 d	9.20 ± 0.56 c	0.93 ± 0.05 c
S+TT2	2.75 ± 0.219 a	0.41 ± 0.022 a	14.65 ± 0.87 a	1.46 ± 0.06 a
